# Spatial expression of fibroblast activation protein-α in clear cell renal cell carcinomas revealed by multiplex immunoprofiling analysis of the tumor microenvironment

**DOI:** 10.1007/s00262-024-03896-y

**Published:** 2025-01-03

**Authors:** Gorka Larrinaga, Miriam Redrado, Ana Loizaga-Iriarte, Amparo Pérez-Fernández, Aida Santos-Martín, Javier C. Angulo, José A. Fernández, Alfonso Calvo, José I. López

**Affiliations:** 1 Department of Nursing, Medicine and Nursing Faculty, University of the Basque Country (UPV/EHU), Barrio Sarriena, S/N, 48940 Leioa, Spain; 2 Department of Physiology, Medicine and Nursing Faculty, University of the Basque Country (UPV/EHU), 48940 Leioa, Spain; 3 Biobizkaia Health Research Institute, 48903 Barakaldo, Spain; 4 Center for Applied Medical Research (CIMA), University of Navarra, IDISNA and Program in Solid Tumors, 31008 Pamplona, Spain; 5 Service of Urology, Basurto University Hospital, 48003 Bilbao, Spain; 6 Clinical Department, Faculty of Medical Sciences, European University of Madrid, 28005 Madrid, Spain; 7 Department of Urology, University Hospital of Getafe, 28907 Madrid, Spain; 8 Department of Physical Chemistry, Faculty of Science and Technology, University of the Basque Country (UPV/EHU), Bº Sarriena, S/N, 48940 Leioa, Spain; 9 Department of Pathology, Anatomy and Physiology, School of Medicine, University of Navarra, CIBERONC, ISCIII, Madrid, Spain, 31009 Pamplona, Spain

**Keywords:** Multiplex immunofluorescence, Fibroblast activation protein, Spatial imaging, Immune cells, Cancer-associated fibroblasts

## Abstract

Clear cell renal cell carcinoma (ccRCC) is one of the most challenging neoplasms because of its phenotypic variability and intratumoral heterogeneity. Because of its variability, ccRCC is a good test bench for the application of new technological approaches to unveiling its intricacies. Multiplex immunofluorescence (mIF) is an emerging method that enables the simultaneous and detailed assessment of tumor and stromal cell subpopulations in a single tissue section. This novel approach represents a promising step forward for analyzing the microenvironmental cell composition and distribution across the tumor and understanding its possible interactions with tumor cells. This study provides the first characterization of the spatial distribution of fibroblast activation protein-α (FAP)-expressing cancer-associated fibroblasts (FAP + CAFs) in conjunction with lymphoid (CD4 + , CD8 + , CD4 + FOXP3 + , and CD20 +) and myeloid (CD68 +) cells in tissue sections from ccRCC in their early phases of evolution (n = 88). Both the tumor center and periphery were analyzed with mIF. FAP + CAFs and tumor-infiltrating lymphocytes (TILs) were significantly concentrated at the tumor periphery. Additionally, elevated percentages of FAP + CAFs were correlated with larger tumors and synchronous metastases. Increased levels of CD68 +  and CD4 + FOXP3 +  cells (above the 75th percentile) were linked to worse cancer-specific survival (CSS) in patients with ccRCC. Furthermore, significant correlations emerged among FAP + CAFs, TILs, and CD68 +  cells, and the co-occurrence of elevated FAP + CAFs, T-cytotoxic (CD8 +), T-regulatory (CD4 + FOXP3 +) cells, and macrophages (CD68 +) at the tumor center were independently associated with worse CSS. These findings suggest that FAP + CAFs contribute to the aggressiveness of ccRCC, and their role is potentially mediated by their ability to foster an immunosuppressive environment within the renal tumor microenvironment.

**Supplementary Information:**

The online version contains supplementary material available at 10.1007/s00262-024-03896-y.

## Introduction

Renal cancer is one of the top ten human neoplasms in Western countries [1] and includes a wide spectrum of sporadic entities linked to a broad range of genetic signatures. Among them, clear cell renal cell carcinoma (ccRCC) is by far the most common subtype, accounting for more than 70% of cases [2]. ccRCC is a major concern because of its aggressiveness and resistance to chemo- and radiotherapy. Cancer-associated mortality, typically in the context of metastatic disease, remains high in patients affected by this tumor type. ccRCC has become a reference tumor in the development of new treatment modalities, which are currently promising for extending life expectancies [3].

Temporal and spatial intratumor heterogeneity (ITH), both in tumor cells and in the tumor microenvironment (TME), is the subject of intense investigation. For example, research has achieved many advances in the knowledge of tumor–nontumor cell interactions [4, 5], tumor evolution [6, 7], metastatic events [8], and mechanisms of therapeutic resistance [9] using this neoplasm as a test bench.

The understanding of the different roles of the microenvironment in modulating tumor behavior has gained momentum in recent years [5]. Previous research has demonstrated that tumor cells and their microenvironment coevolve temporally and spatially within the same neoplasm [10]. Recent studies using thorough renal tumor sampling have revealed how tumor cells evolve specifically in response to local microenvironmental pressures; for example, metastasizing capacities differ significantly at the tumor’s center versus its periphery [11], and the spatial patterns of ITH and tumor cell distribution are distinct [12]. The importance of deciphering the varied ITH patterns of renal tumor cells and their microenvironment warrants the implementation of a rational tumor sampling protocol in routine practice [13–15].

The interaction between cancer-associated fibroblasts (CAFs), a major component of the tumor stroma, and immune cells within the TME is a pivotal determinant of cancer progression and therapeutic response [16, 17]. CAFs secrete various cytokines, growth factors, and chemokines that contribute to the recruitment and polarization of immune cells, fostering an immunosuppressive environment [16]. CAFs abundantly express fibroblast activation protein-α (FAP), which stands at the forefront of these intricate biological phenomena [17, 18]. This glycoprotein modulates the extracellular matrix composition and stromal cell interactions through enzymatic and non-enzymatic mechanisms, collectively fueling an environment conducive to tumor progression and treatment resistance [17, 18]. The unique feature of FAP, which is predominantly absent from most normal tissues under physiological conditions, underscores its potential as a highly promising target for FAP-specific therapies and cutting-edge molecular imaging techniques [18–21].

The development of multiplex image analysis techniques has marked the onset of an era with the potential to transform pathological diagnosis and precision medicine in cancer treatment [22]. These methods enable the simultaneous and detailed assessment of multiple biomarkers, providing researchers with a comprehensive understanding of spatial interactions throughout the tumor ecosystem. The studies published thus far on ccRCC suggest a heterogeneous distribution of several stromal cell subpopulations within the tumor tissue, with implications for prognosis and treatment response [23–30].

Our experience in the qualitative analysis of FAP by immunohistochemistry (IHC) [31–33], along with that of other authors [34, 35], has revealed a striking association between high FAP expression and early metastatic events as well as poor patient survival and an inadequate response to anti-angiogenic treatment. To comprehensively understand the quantitative spatial distribution of FAP + CAFs and their association with immune cells (T and B lymphocytes and macrophages) as well as their clinicopathological significance, we conducted multiplex immunofluorescence (mIF) analysis in formalin-fixed paraffin-embedded (FFPE) samples obtained from a cohort of 88 patients with ccRCC with over 5 years of clinical follow-up.

## Methods

### Patients and samples

Tumor tissues from 88 patients with ccRCC who were surgically treated at Basurto University Hospital between 2012 and 2016 were collected for this study. The inclusion criteria were 1) adult patients (age > 18 years), 2) tumors obtained by partial or radical nephrectomy and 3) pathologically diagnosed as ccRCC. Patients with preoperative systemic treatment and without complete clinical and pathological data were excluded from the study. Tumor selection for the study focused preferentially, but not exclusively, on organ-confined tumors less than 7 cm in diameter, without tumor necrosis or metastatic spread, with the aim of investigating the tumor microenvironment in the early stages of tumor development. Representative samples of the tumor center and periphery were included in tissue microarrays (TMAs) for further mIF analysis. TMAs were built with cores with a diameter of 2.5 mm obtained from optimally preserved regions of the tumor center and periphery. Clinical follow-up of patients extended from ccRCC diagnostic data to January 31, 2023.

### Multiplex immunofluorescence analysis

The Opal 7 Solid Tumor Immunology (OP7TL4001KT) Kit from Akoya (California, USA) was used for multispectral staining in seven TMAs, following the manufacturer’s instructions. Briefly, slides were deparaffinized and hydrated, and sequential immunolabeling of CD4 + (T helper cells), CD8 + (T cytotoxic cells), CD4 + FOXP3 + (T-regulatory cells), CD20 + (B lymphocytes), CD68 + (macrophages), and Pan-CK + (tumor cells) was performed with appropriated antigen retrieval conditions and dilutions (see details in Supplementary Table S1). A second panel of multispectral staining was developed by exchanging anti-CD-20 for anti-FAP (207,178, Abcam, Cambridge, UK) antibodies (see details in Supplementary Table S1). Thorough controls were performed for FAP immunostaining and immunofluorescent labeling in the multiplex analysis, as the rest of the markers have been extensively validated by Akoya and the panel is commercially available. Controls included removal of primary antibody, secondary antibody or the fluorescent Opal, as well as elimination of tissue autofluorescence (described at the Results section). For sample scanning, spectral unmixing, and signal quantification, a Vectra Polaris Automated Quantitative Pathology Imaging System (Akoya) was used. Data were extracted and analyzed with Phenochart, InForm 2.4 (Akoya), and QuPath (open-source software for digital pathology image analysis). Data are presented as the number of each particular phenotype with respect to the total number of cells for each case (based on DAPI staining).

### Statistical analysis

Statistical analysis was conducted with SPSS® 28.0 software. To assess the normality of the data distribution, the Kolmogorov–Smirnov test was applied. Subsequently, data were subjected to either parametric or nonparametric tests depending on the normality assessment.

The correlation between biomarker percentages and other quantitative variables was evaluated with the Spearman’s Rho test. When comparing percentages between two groups or more than two groups, a Mann–Whitney U (Mann-U) test or Kruskal–Wallis test was used, respectively. Categorical variable comparisons were conducted with the Chi-square (χ2) test.

To assess the association between biomarkers and cancer-specific survival (CSS) in patients with ccRCC, Kaplan–Meier curves and log-rank tests were used. Finally, to evaluate the independent effects of biomarkers or pathological variables on CSS, multivariate analyses were conducted by employing the Cox regression model with the backward Wald method.

## Results

### Demographic and pathological data

Most of the patients were male (60 males and 28 females), and the average age was 61.7 years (range 36–82). Patients were followed for a mean of 88.7 months (range 2–132). When the follow-up ended in January 2023, 62 patients were still alive, 20 had died of disease, and 6 had died of other causes. The tumor diameter was  ≤ 7 cm in 67 cases (76.1%) and > 7 cm in 21 cases (23.9%). The Fuhrman grade distribution was as follows: G1, 3 cases (3.4%); G2, 45 cases (51.2%); G3, 30 cases (34.1%); and G4, 10 cases (11.3%). Tumors were organ-confined in 70 cases (79.5%) ((pT1, 59 cases (67%); pT2, 11 cases (12.5%)) and non-organ-confined in 18 cases (20.5%) (pT3, 16 cases (18.1%); pT4, 2 cases (2.2%)). Lymph node invasion (pN) was detected in 6 cases (6.8%), and distant metastases (pM) were found in 10 cases (11.3%). The main pathologic data are summarized in Table 1.

**Table 1:** Quantitative expression of FAP, CD4, CD8, CD4 + FOXP3 + , CD20, and CD68 positive cells in terms of biological aggressiveness

Pathological parameters	C		P		C		P		C		P		C		P		C		P		C		P	
	FAP	p =	FAP	p =	CD4	p =	CD4	p =	CD8	p =	CD8	p =	CD20	p =	CD20	p =	CD4 + FOXP3 +	p =	CD4 + FOXP3 +	p =	CD68	p =	CD68	p =
*Fuhrman Grade (G)*																								
Low (G1-G2) (n = 48)	23.6	0.62	32.6	0.27	2.5	0.37	4.1	**0.03**	1.4	0.08	2.4	**0.01**	0.39	0.21	1.3	0.27	0.15	0.85	0.25	0.25	0.9	0.32	0.87	**0.05**
High (G3-G4) (n = 40)	30		40.0		2.9		5.7		3.3		5.8		0.13		2.5		0.2		0.38		0.96		1.6	
*Necrosis*																								
No (n = 64)	23.4	0.15	32.8	0.11	2.3	**0.008**	4.2	**0.023**	1.4	0.06	2.5	**0.005**	0.21	0.59	1.6	0.78	0.16	0.97	0.23	**0.03**	0.91	0.11	1.1	0.1
Yes (n = 24)	34.2		44.8		3.8		6.4		4.4		8.1		0.43		2.7		0.24		0.46		1.0		1.5	
*Diameter*																								
≤ 7 cm (n = 67)	24.1	0.1	32.6	**0.02**	2.4	0.06	4.5	**0.05**	1.5	**0.01**	3.0	**0.006**	0.28	0.38	1.6	0.07	0.16	0.07	0.28	0.18	0.82	**0.009**	1.1	**0.03**
> 7 cm (n = 21)	34.5		48.0		3.6		6.2		4.8		7.2		0.23		2.9		0.26		0.41		1.26		1.8	
*Local Invasion (pT)*																								
pT1-pT2 (n = 70)	26.0	0.73	36.1	0.97	2.7	0.97	4.7	0.35	2.1	0.63	3.5	0.12	0.31	0.12	1.9	0.44	0.18	0.4	0.29	0.75	0.91	0.9	1.2	0.37
pT3-pT4 (n = 18)	28.5		36.7		2.6		5.5		2.7		6.4		0.15		2.0		0.18		0.38		0.99		1.6	
*Lymph node invasion*																								
No (n = 82)	25.8	0.33	35.3	0.29	2.7	0.48	5	0.7	2.1	0.52	3.7	0.6	0.28	0.51	1.9	0.92	0.17	0.18	0.3	0.3	0.46	**0.04**	1.2	**0.03**
Yes (n = 6)	37.6		48.1		1.8		3.6		5.0		9		0.19		1.9		0.26		0.46		0.9		2.1	
*Distant Metastasis*																								
No (n = 78)	25.1	0.3	34.2	**0.04**	2.6	0.28	4.8	0.53	1.9	**0.04**	3.5	0.21	0.27	0.18	1.9	0.51	0.15	0.07	0.29	0.09	0.8	**0.002**	1.1	**0.003**
Yes (n = 10)	37.7		51.2		3.1		5.3		4.9		7.8		0.28		2.0		0.36		0.46		1.9		2.1	

Values represent relative percentage (%) of biomarker-positive cells in relation to the total number of cells in each sample, both at the center (C) and at the periphery (P) of the tumor. Groups were compared by Mann-U test. Significant p values are highlighted in bold. pT1-pT2 = organ-confined, pT3-pT4 = non-organ-confined

### Quantitative expression of FAP+, CD4+, CD8+, CD4+FOXP3+, CD20+, and CD68+ cells in terms of biological aggressiveness

The results of the quantitative analyses are expressed as the percentage of biomarker-positive cells relative to the total number of cells in each sample. Several immunohistochemistry and multiplex immunofluorescence controls were included to ensure the specificity of the immunolabeling, especially for FAP, which was not included in the Akoya kit: omission of the primary antibody (Supplementary Figure S1 A-D), omission of the secondary antibody or the Opal fluorochrome (Supplementary Figure S1 E) and single channel analysis to confirm lack of fluorochrome overlap (Supplementary Figures S2 and S3).

**Fig. 1:**
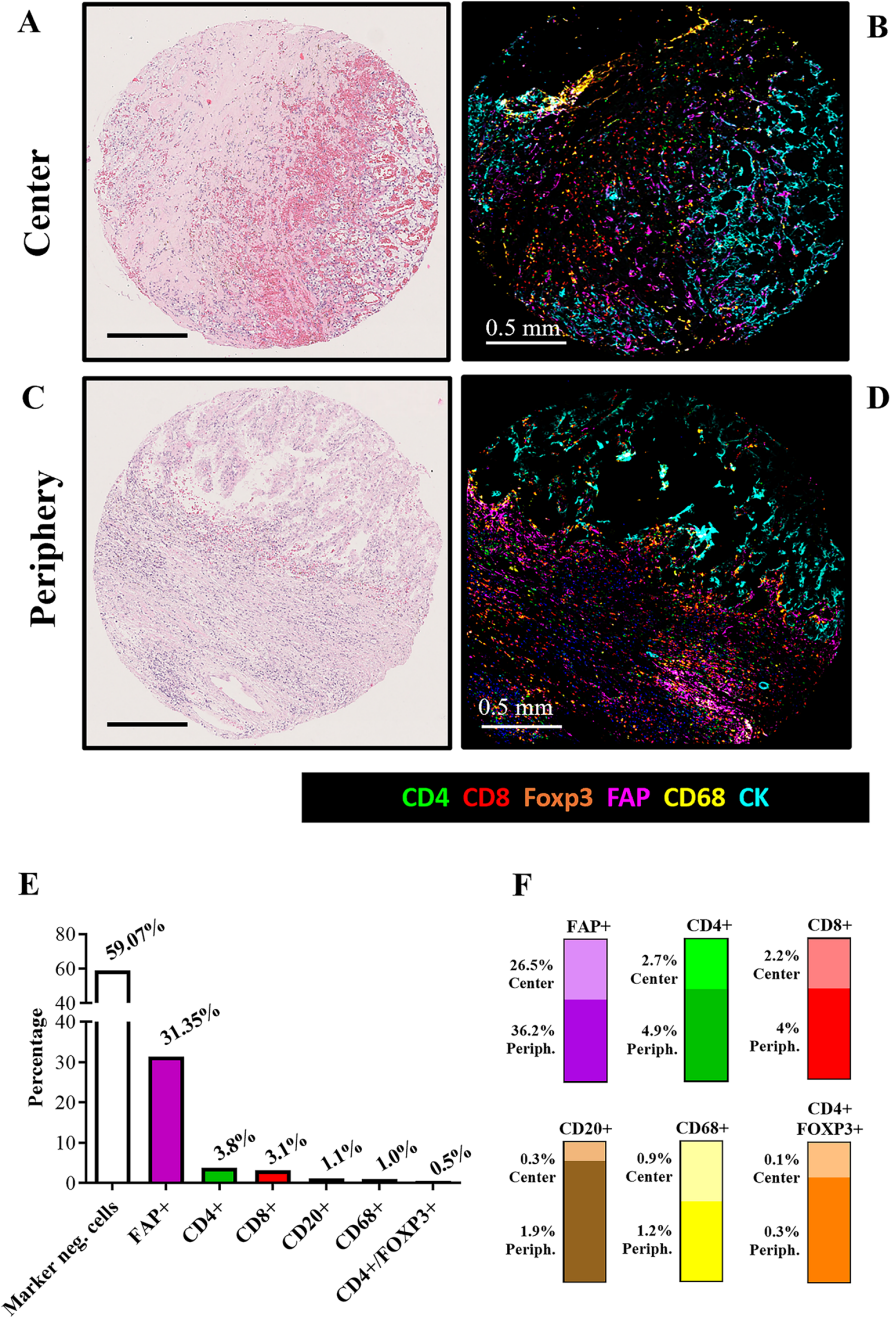
Hematoxylin and eosin (A, C) and Multiplex immunofluorescence analysis (mIF) of CAFs (FAP +), T-helper (CD4 +), T-cytotoxic (CD8 +) and T-regulatory (CD4 + FOXP3 +) lymphocytes, macrophages (CD68 +), and tumor cells (pan-CK +) at the center (A and B) and the periphery (C and D) of clear cell renal cell carcinomas (ccRCCs). Percentages of different stromal and tumor cells obtained through mIF are displayed (E, F)

Overall, FAP +  cells were the most abundant stromal cells in the tumor (Fig. 1). CD4 +  and CD8 + T-lymphocytes were the second and third most abundant cells, respectively. The presence of CD68 + (macrophages) and CD20 + (B-lymphocytes) was detected in lower amounts. Finally, CD4 + FOXP3 + (T-regulatory) cells were the least abundant cell population in the tumor (Fig. 1 and Supplementary Figure S4).

**Fig. 2:**
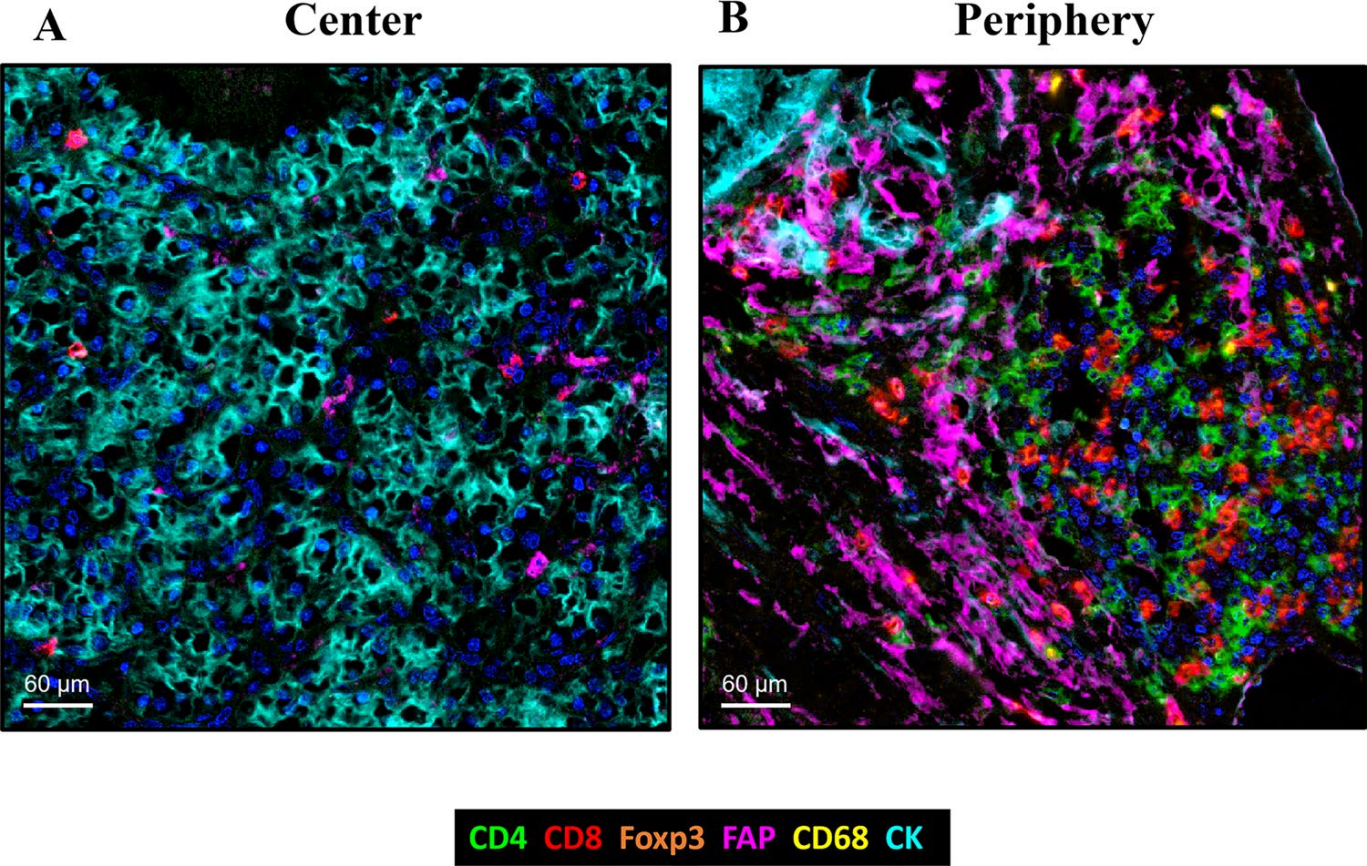
Quantitative expression of FAP + , CD4 + , CD8 + , CD4 + FOXP3 + , and CD68 +  cells at the tumor center (**A**) and periphery (**B**) of ccRCC tissues. FAP-expressing CAFs (FAP + CAFs), T-helper, T-cyt, and T-reg lymphocytes were significantly more abundant at the tumor periphery than at the center

The spatial distribution of these cells in the tumor showed a significantly higher percentage of FAP + (26.5% at center vs. 36.2% at periphery, Mann-U *p* = 0.003), CD4 + (2.7% vs. 4.9%, *p* < 0.001), CD8 + (2.2% vs. 4%, *p* < 0.001), CD20 + (0.3% vs. 1.9%, *p* < 0.001), and CD4 + FOXP3 +  cells (0.18% vs. 0.31%, *p* < 0.001) at the tumor periphery (Figs. 1, 2 and Supplementary Figure S5). By contrast, the distribution of CD68 +  cells was similar at the center and periphery (0.93% vs. 1.24%, *p* = 0.65).

On the other hand, elevated percentages of FAP +  cells exhibited significant positive correlations with higher percentages of CD4 + (tumor center, Spearman’s Rho = 0.24, *p* = 0.026; tumor periphery, Spearman’s Rho = 0.237; *p* = 0.03), CD8 + (tumor center, Spearman’s Rho = 0.524, *p* < 0.001; tumor periphery, Spearman’s Rho = 0.374; *p* < 0.001), CD4 + FOXP3 + (tumor center, Spearman’s Rho = 0.486, *p* < 0.001; tumor periphery, Spearman’s Rho = 0.281; *p* = 0.01), and CD68 +  cells (tumor center, Spearman’s Rho = 0.239, *p* = 0.237; tumor periphery, Spearman’s Rho = 0.528, *p* < 0.001). However, we did not observe a correlation between FAP +  and CD20 +  cells (tumor center, Spearman’s Rho = 0.11, *p* = 0.31; tumor periphery, Spearman’s Rho = 0.03; *p* = 0.8).

### Quantitative expression of FAP, CD4, CD8, CD4 + FOXP3 + , CD20, and CD68 positive cells in terms of biological aggressiveness

The stratification of clinical pathological parameters with prognostic implications included the Fuhrman grade, tumor phenotype, presence of necrosis, tumor diameter, local invasion (pT), nodal involvement (pN), and distant metastases (pM). We assessed whether the percentages of each biomarker varied according to the sex and gender of the patients to avoid bias. The results did not show any correlations (Spearman’s Rho, *p* > 0.05 in all cases).

The observed trend indicates that CAFs expressing FAP as well as CD68 +  macrophages and tumor-infiltrating lymphocytes (TILs) (CD4, CD8, and CD4 + FOXP3 +) are more abundant in the more aggressive ccRCCs. The results are summarized in Table 1.

*Fuhrman grade*. At the tumor periphery, high-grade ccRCCs showed significantly higher percentages of CD4 +  and CD8 +  cells than low-grade ones. CD68 +  cells were also more abundant, although this result was almost significant (*p* = 0.05).

*Tumor necrosis*. A total of 24 ccRCCs showed tumor necrosis. All of the cells studied were more abundant in the cases that showed necrosis. This difference was statistically significant for CD4 + , CD8 + , and CD4 + FOXP3 +  cells at the tumor periphery and for CD4 +  at the tumor center.

*Tumor diameter*. The results were stratified into two groups: pT1 (≤ 7 cm) and pT2 (> 7 cm). ccRCCs larger than 7 cm displayed significantly higher percentages of FAP + , CD8 + , and CD68 +  cells at the tumor periphery. On the other hand, CD8 +  and CD68 +  cells were also more abundant at the center of ccRCCs larger than 7 cm.

*Local invasion (pT)*. This pathological parameter was stratified as organ-confined (pT1-pT2) and non-organ-confined (pT3-pT4) tumors, and the results did not show significant differences.

*Lymph node invasion (pN).* ccRCCs with loco-regional lymph node invasion showed significantly higher percentages of CD68 +  cells than tumors without node invasion at the tumor center and periphery.

**Fig. 3:**
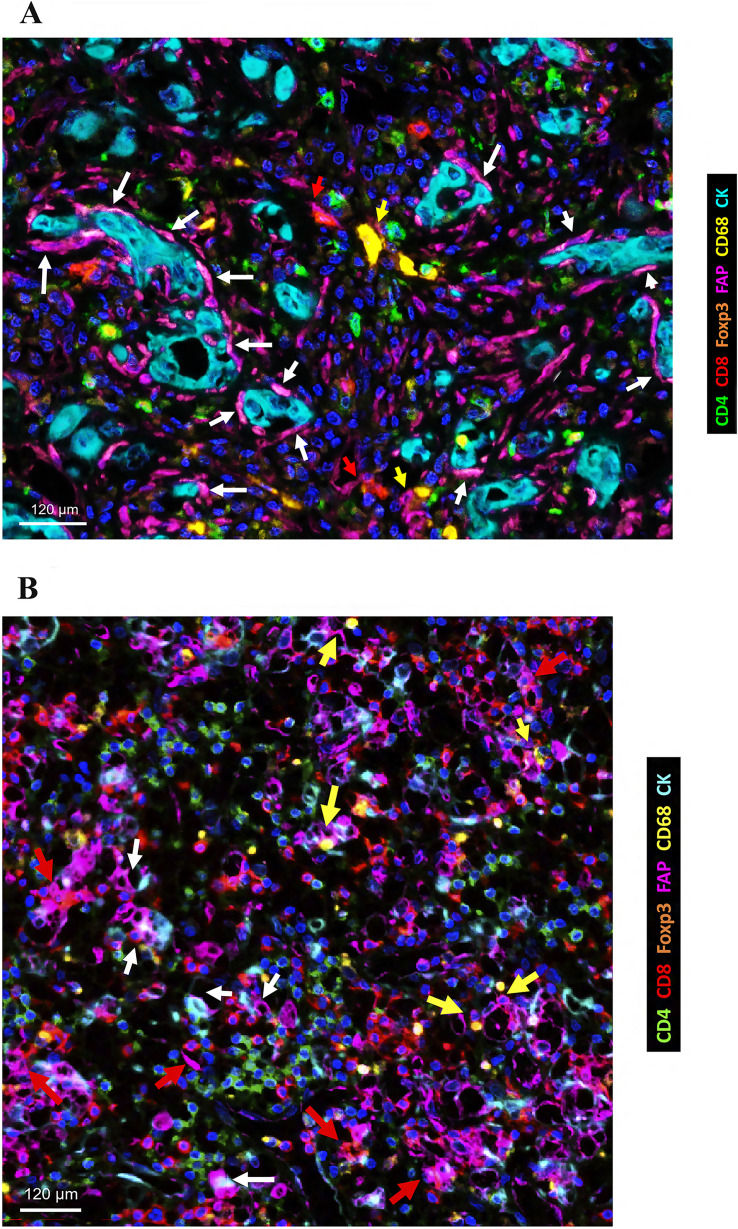
Quantitative expression of stromal cells in ccRCCs with synchronous distant metastasis. Multiplex immunofluorescence (mIF) analysis revealed that tumors debuting with distant metastases had higher concentrations of FAP + CAFs, T-cyt and T-reg lymphocytes, and macrophages. The figure shows the distribution of FAP+, CD8+, and CD68+ cells at the center (**A**) and the periphery (**B**) in the primary tumor of a metastasized ccRCC. FAP + CAFs (white arrows) show a spindle morphology when surrounding tumor nests near CD8 (red arrows) and CD68 (yellow arrows) cells. The spindle morphology is accentuated at the tumor center because of the associated sclerosis in the stroma

*Distant metastasis (pM).* ccRCCs with synchronous distant metastases exhibited significantly higher percentages of FAP +  and CD68 +  cells at the tumor periphery as well as elevated percentages of CD8 +  and CD68 +  cells at the tumor center. Furthermore, in these aggressive tumors, we observed FAP +  stromal cells surrounding tumor cells and near CD8 +  and CD68 +  cells (Fig. 3).

### Quantitative expression of FAP + , CD4 + , CD8 + , CD4 + FOXP3 + , CD20 +  and CD68 +  cells in terms of Cancer-Specific Survival

The quantitative data were classified into percentiles to find cutoff points for the survival analysis. Because higher percentages of all biomarkers were associated with aggressive behavior, the 50th percentile (P50) and 75th percentile (P75) were considered the optimal cutoff points for each biomarker. The values for P50 and P75 and the log-rank test p values of each survival analysis are provided in Table 2.

**Table 2:** Log-rank test p values of cancer-specific survival (CSS) by percentiles 50 (P50) and P75

Percentile (P)	Center	Periphery	Center	Periphery	Center	Periphery	Center	Periphery	Center	Periphery	Center	Periphery
	FAP	FAP	CD4	CD4	CD8	CD8	CD20	CD20	CD4 + FOXP3 +	CD4 + FOXP3 +	CD68	CD68
P50	18.763	33.651	1.746	3.325	0.687	1.744	0.066	0.501	0.081	0.201	0.527	0.518
P75	36.735	53.799	3.732	7.227	2.032	4.872	0.173	2.038	0.266	0.437	0.159	1.712
	Log-rank *p*=	Log-rank *p*=	Log-rank *p*=	Log-rank *p***= **	Log-rank *p***= **	Log-rank *p***= **	Log-rank *p***= **	Log-rank *p***= **	Log-rank *p***= **	Log-rank *p***= **	Log-rank *p***= **	Log-rank *p***= **
< versus ≥ P50	0.9	0.43	0.89	0.79	0.34	0.41	0.7	0.77	0.2	0.46	**0.003**	0.39
< versus ≥ P75	0.89	0.36	0.84	0.93	0.44	0.8	0.84	0.75	**0.029**	0.97	**0.001**	**0.027**

Numbers represent the relative percentage (%) of biomarker-positive cells, both at the center and at the periphery of the renal tumor. P50 and P75 were selected as cutoff values of each biomarker for survival analyses. Significant results of the log-rank test (*p* < 0.05), highlighted in bold, correspond to equal or higher percentages than P50 and P75 of each biomarker, which significantly associated with worse CSS

**Fig. 4:**
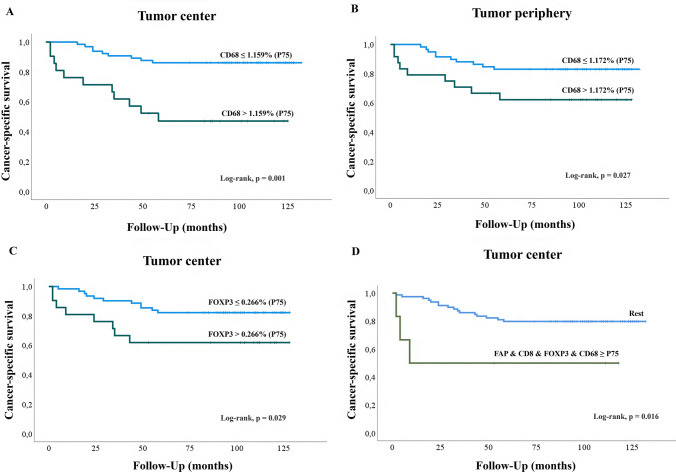
Kaplan–Meier curves illustrating the association between cancer-specific survival (CSS) of patients with ccRCC and quantitative expression of FAP + CAFs, T-cyt and T-reg lymphocytes, and macrophages. High percentages (above the 75th percentile (P75)) of macrophages at the center (**A**) and the periphery of tumors (**B**) were associated with poor CSS. High levels (above P75) of CD4 + FOXP3 +  cells (T-reg) were also associated with worse CSS (C). The expression of FAP + CAFs and T-cyt, T-regs, and macrophages at the tumor center (all above the P75 threshold) was associated with worse CSS (D). (Rest = tumors with all other combinations, i.e., all cases in which the four biomarkers did not coincide above the P75 threshold)

*Kaplan–Meier curves and log-rank test*. Values equal to or higher than the P75 values for CD68 +  at both locations and for CD4 + FOXP3 +  at the tumor center were significantly associated with worse cancer-specific survival (CSS), as shown by the Kaplan–Meier curves in Fig. 4. Similarly, values equal to or higher than the P50 values for CD68 +  at the tumor center were also associated with worse CSS (Table 2).

Given that high values for FAP + , CD8 + , CD4 + FOXP3 + , and CD68 + were associated with the presence of synchronous metastasis and/or worse CSS when analyzed individually, we conducted a combined analysis of these four biomarkers (using P50 and P75 as references) and their association with patients’ survival. Interestingly, the presence of both FAP + CAFs, T-cyts (CD8 +), T-regs (CD4 + FOXP3 +), and macrophages (CD68 +) above P75 (log-rank *p* = 0.016) at the tumor center was associated with worse CSS (Fig. 4). We also created double and triple combinations, and results of the corresponding log-rank test are summarized in Supplementary Table S2.

*Univariate and multivariate Cox regression analyses.* Univariate analysis was performed to test the individual association of each biomarker and pathological variable with patient’s survival (Supplementary Table S3). The univariate Cox regression model showed that high grade (G1-G2 *vs.* G3–G4, *p* = 0.006), large tumor diameter (≤ *vs.* > 7 cm, *p* = 0.001), non-organ-confined tumors (pT1-pT2 *vs.* pT3–pT4, *p* = 0.002), lymph node invasion (*p* = 0.001), distant metastasis (*p* = 0.001), high densities of CD68 +  cells (≥ P75 at tumor center, *p* = 0.001; and tumor margin, *p* = 0.03; and P50 at the tumor center, *p* = 0.006), and CD4 + FOXP3 +  cells (≥ P75 at the tumor center, *p* = 0.037) were associated with worse CSS.

These variables were included in the multivariate Cox regression analysis to determine whether the quantitative expression of each stromal biomarker was an independent prognostic factor for CSS (Table 3). To avoid mathematical bias in the regression model, tumor diameter was not included in the analysis because the local invasion (pT) accounts for this variable. The logistic model, resulting from a backward Wald stepwise elimination of variables, revealed that local invasion, lymph node and distant metastasis, and values higher than the P50 for CD68 + (*p* = 0.046) at the tumor center were independent prognostic factors for CSS. Percentages of T-regs and macrophages above the P75 threshold also appeared in the final step of the backward stepwise method, but they did not reach statistical significance (*p* = 0.063 and *p* = 0.086 respectively).

**Table 3:** Predictive model (Cox regression) for cancer-specific survival (CSS) prediction by each biomarker and pathological variables in ccRCC patients

3A									
CSS	Variables	Tumor center				Tumor periphery			
		*p* =	ExpB	Inf	Sup	*p* =	ExpB	Inf	Sup
Multiple Cox Regression	CD68 (P75)	0.063	3.47	0.94	9.44	0.246	1.84	0.66	5.18
	Grade	0.296	1.09	0.57	6.28	0.509	1.57	0.41	5.94
	pT	0.084	2.98	0.89	6.06	0.075	2.63	0.91	7.63
	pN	0.088	2.91	0.85	10.08	0.2	2.34	0.64	8.58
	pM	0.17	1.88	0.66	10.84	0.004	6.96	1.74	17.35
Final Step of Wald Method	**CD68 (P75)**	0.086	2.96	0.87	8.32	–-			
	**pT**	**0.049**	3.86	1.00	6.62	**0.048**	2.64	1.01	6.93
	**pN**	0.066	3.37	0.92	11.32	0.078	3.02	0.88	10.31
	**pM**	0.052	3.76	0.99	13.55	**0.001**	6.96	2.42	20.01

3B					
CSS	Variables	Tumor center			
		*p* =	ExpB	Inf	Sup
Multiple Cox Regression	FOXP3 (P75)	0.147	2.21	0.76	6.48
	Grade	0.638	1.35	0.39	4.70
	pT	0.061	2.81	0.95	8.27
	pN	0.18	2.43	0.66	8.95
	pM	0.004	5.72	1.74	6.45
Final Step of Wald Method	**FOXP3 (P75)**	0.063	2.66	0.95	7.44
	**pT**	**0.026**	3.29	1.16	9.36
	**M**	**0.001**	8.12	2.94	22.46

3C					
CSS	Variables	Tumor center			
		*p* =	ExpB	Inf	Sup
Multiple Cox Regression	CD68 (P50)	0.074	2.91	0.9	9.4
	G	0.32	1.9	0.53	6.85
	pT	0.071	2.48	0.92	6.68
	pN	0.197	2.19	0.67	7.18
	pM	0.008	4.51	1.49	13.63
Final Step of Wald Method	**CD68 (P50)**	**0.046**	3.27	1.02	10.46
	**pT**	**0.035**	2.95	1.08	8.03
	**M**	**0.001**	6.52	2.23	19.0

3D					
CSS	Variables	Tumor center			
		*p* =	ExpB	Inf	Sup
Multiple Cox Regression	FAP/CD8/FOXP3/CD68 (P75)	0.174	3.02	0.61	14.9
	Grade	0.189	2.44	0.65	9.25
	pT	0.269	1.79	0.64	5.05
	pN	0.21	2.35	0.62	8.9
	pM	0.005	5.23	1.66	16.8
Final Step of Wald Method	**FAP/CD8/FOXP3/CD68 (P75)**	**0.035**	4.77	1.11	20.5
	**Grade**	0.067	3.32	0.92	12.0
	**M**	**0.001**	6.68	2.26	19.7

Selected independent variables were Percentile 75 (P75) cutoff value for CD68 (**A**) at both locations of the tumor, P75 for FOXP3 (**B**) at the tumor center, P50 for CD68 at the tumor center (**C**), and P75 for quadruple combination of FAP/CD8/FOXP3/CD68 (**D**); and pathological variables such as Fuhrman grouped grade (low *vs.* high), grouped local invasion (pT, confined *vs.* not-confined), lymph node invasion (pN, no *vs.* yes), and distant (pM, no *vs.* yes) metastases. ExpB with confidence interval (CI, inferior and superior) is also included. Variables resulting from the backward Wald stepwise method are highlighted in bold. (*) To avoid confusion, in this table T-regulatory cells (CD4 + FOXP3 +) are described as FOXP3 only.

Multivariate analysis was also conducted using variables created by combining biomarkers. The Cox regression model revealed that densities of both FAP + CAFs, T-cyts, T-regs, and macrophages over the P75 at the tumor core were independent prognostic factors for worse CSS (*p* = 0.035) (Table 3). The results of the multivariate analysis with double and triple combinations are described in Supplementary Table S4.

## Discussion

mIF is a recently developed tool that represents a step forward in the analysis of tumor and stromal cell subpopulations in a single tissue section [22]. Over the past few years, renal cancer researchers have increasingly adopted this technique. The extensive existing knowledge about immune cell subpopulations in the current era of cancer immunotherapy has directed the studies toward detecting biomarkers of inflammatory cells [23–30]. However, an increasing number of studies have unveiled a plethora of distinct CAF subpopulations and highlighted their pivotal role in tumor biology [16, 17, 36]. This growing body of evidence underscores the need to include CAFs in multiplex analyses to comprehensively explore the TME [37, 38]. Our study addresses this demand and provides the first characterization of the spatial distribution of FAP + CAFs in conjunction with both lymphoid and myeloid cells in ccRCC tissue sections.

FAP is a biomarker present in the most abundant subpopulations of CAFs of several solid tumors [16, 17]. According to the recent classification proposed by Cords et al. [36], matrix, inflammatory, tumor-like, antigen-presenting, and dividing CAFs, collectively referred to as myofibroblastic CAFs (myCAFs), express this protein. Qualitative analyses of ccRCC tissues with IHC have demonstrated that FAP expression is indicative of poor prognosis [31–34] and limited response to anti-angiogenic treatment with tyrosine-kinase inhibitors (TKIs) [35]. Furthermore, myCAFs expressing *FAP* mRNA are more abundant in ccRCCs that are resistant to immune checkpoint inhibitors (ICIs) [30]. The results of our mIF-based quantitative analysis were consistent with these findings; the percentage of FAP + CAFs was higher in the most aggressive tumors, particularly those with larger diameters and those presenting with metastases at diagnosis. Together, this evidence underscores the pivotal role of FAP as a prognostic biomarker associated with unfavorable treatment responses in ccRCC, emphasizing the importance of considering this biomarker in shaping precision medicine strategies for this disease [16, 18].

One of the advantages of multiplex imaging techniques over single-cell sequencing methods is the ability to analyze tissue while preserving its architectural integrity [22]. As a result, these methods offer valuable spatial insights into ITH, of which ccRCC is a paradigmatic example [6, 7]. Our study revealed a higher percentage of FAP + CAFs at the tumor periphery, in agreement with the findings of Davidson et al. [30], who stained myCAFs with the protein transgelin and observed a similar spatial distribution. Furthermore, we observed a higher percentage of TILs at the tumor margins, corroborating the findings of previous mIF studies that have mapped the immune landscape of ccRCC [23, 24, 26, 29].

The mechanism through which this heterogeneous spatial distribution influences the progression of renal tumors is far from being fully understood. Our quantitative analysis revealed a significant association between the abundance of FAP + CAFs at the tumor’s periphery and the onset of metastasis. Nevertheless, we also observed a trend toward higher percentages of these fibroblasts at the center of more aggressive tumors, in line with previous IHC studies that have suggested that FAP + CAFs play a key role not only at the tumor’s margin but also at its core [31–33].

Similarly, the mean percentages of TILs were higher at the tumor periphery, but more aggressive ccRCCs also exhibited increased percentages of these lymphoid cells at the core. Unlike many solid tumors, the presence of T-cyt (CD8 +) infiltrates in ccRCC tissues is associated with an unfavorable prognosis [24]. Nevertheless, despite the abundance of these TILs at the tumor margins [23, 24, 26], a large presence of tumor-specific (CD39 +) and exhausted (PD-1 +) T-cyt cells in the tumor core could be a determinant of ccRCC progression [24]. The association between elevated T-reg cell percentages (CD4 + FOXP3 +) at the tumor core and worse CSS in patients in our study might also indicate the existence of an intratumoral immunosuppressive environment that allows tumor cells to evade immune surveillance.

Regarding tumor-associated macrophages (TAMs), a recent study found that the geospatial clustering of CD163 + M2-polarized macrophages within the stromal compartment at the renal tumor margins was associated with poor clinical outcomes [26]. Our data partially agree with these findings, as the abundance of macrophages (CD68 +) was associated with ccRCC aggressiveness and worse CSS, with similar associations observed at the tumor center and periphery. Additional research is required to determine whether M2 macrophages are also present in the central regions of these tumors and whether other phenomena or interactions between TAMs and the TME contribute to more severe disease progression.

The average density of TAMs in tumor tissue was lower than that reported by other studies [39–41]. One hypothesis to explain this discrepancy is that our series primarily consists of ccRCCs in an early stage of evolution, as they were predominantly organ-confined, had smaller diameters, and lacked tumor necrosis and metastatic spread. It is well known that TAM infiltrates are higher in the stroma of advanced ccRCCs [42, 43]. For instance, in mIF analyses, Hajiran et al. [44] and Chakiryan et al. [26] confirmed this, showing higher TAM densities in ccRCCs with poorer prognostic pathological variables, such as larger tumor size, presence of metastases, and reduced survival [26, 44]. The average TAM density in their studies was higher than ours because their series primarily included advanced ccRCCs. Therefore, these differences in TAM densities may be related to the evolutionary stage of the primary tumor.

Our analysis further unveiled that the co-occurrence of high percentages of FAP + CAFs, T-cyt, T-reg, and macrophages (above the P75 threshold) at the center of the primary tumor was significantly associated with worse CSS. Additionally, the abundance of FAP + CAFs was proportionally correlated with that of lymphoid and myeloid cells in both tumor regions. Furthermore, in the most aggressive tumors in which these TME cells were abundant, we observed FAP + CAFs surrounding tumor cells (as previously reported with myCAFs in other solid tumors [16]) as well as near CD8** + **and CD68** + **cells.

One of the most challenging aspects to understand is the cross-talk occurring between different cells within the TME and how this affects tumor cells’ acquisition of invasive properties. Relevant insights have emerged from single-cell transcriptome sequencing and multiplex imaging studies, contributing to our understanding of this phenomenon. For instance, the abundance of exhausted T-cyt cells (CD8 + CD39 + PD-1 +) in ccRCC was found to be positively correlated with the abundance of T-regs (CD4 + FOXP3 +) and PD-L1 +  tumor cells [24]. Furthermore, it was demonstrated that M2 macrophages were associated with these exhausted T-cyt cells [26], which, in turn, are linked to mutations in *BAP1* within tumor cells [29].

The mechanisms through which CAFs contribute to the establishment of these immunosuppressive environments are diverse. Consistent with our findings, a previous study reported that M2 macrophages and T-regs were more abundant in microenvironments rich in CAFs [16]. Through the secretion of different cytokines and chemokines (such as IL-6, IL-8, TGFβ, and CXCL-12), CAFs can induce the differentiation of these immunosuppressive cells and a decrease in the cytotoxic capacity of CD8** + **cells [16, 17, 45, 46]. Additionally, myCAFs establish fibrotic niches at the periphery of renal tumors and associate with mesenchymal-like tumor cells, which display a more aggressive phenotype [30]. The remodeling actions of CAFs on the extracellular matrix limit the access of tumor-killing immune cells and hinder the penetration of therapeutic agents [16]. Experiments involving FAP overexpression or depletion have consistently resulted in the induction of these immunosuppressive phenomena [16, 29]. Furthermore, FAP + CAFs are enriched in tumors of patients who do not respond to ICI treatments [20, 47]. Thus, both preclinical and clinical investigations indicate that FAP is a promising candidate for histopathological imaging, advanced radiodiagnostics, and emerging therapeutic modalities [18–21].

In this study, FAP was employed as the primary biomarker to identify CAFs. Although the main source of FAP is fibroblasts [20], its expression is not confined solely to these cells and can also be detected in other cell types, including mesenchymal-like tumor cells, which may not consistently express cytokeratin (CK) [48]. This raises the possibility that some FAP +  cells identified in our analysis could originate from the tumor itself rather than from the stromal compartment. While FAP remains a valuable marker for exploring stromal dynamics within the tumor microenvironment, relying on it alone may not adequately distinguish the CAF population. Future studies incorporating additional fibroblast markers [17, 18, 20], such as α-smooth muscle actin (α-SMA) or fibroblast-specific protein-1 (FSP-1), in conjunction with epithelial and mesenchymal markers, would offer a more comprehensive characterization of the cell types involved and their contribution to ccRCC development and progression.

In summary, our study presents a comprehensive analysis of the spatial distribution of FAP + CAFs and inflammatory cells in ccRCC, elucidating their heterogeneous presence. Notably, FAP + CAFs and TILs displayed a pronounced presence at the tumor periphery, which suggests that they play different roles within the TME. Moreover, higher levels of FAP + CAFs were associated with larger tumors and synchronous metastases, whereas increased CD68 +  and CD4 + FOXP3 +  cells (above P75) were correlated with heightened aggressiveness and reduced survival rates. This study also revealed significant correlations between FAP + CAFs, TILs, and CD68 +  cell densities, highlighting their intricate relationships. Additionally, the co-occurrence of elevated FAP + CAFs, T-cyt, T-reg, and macrophages at the tumor center was independently linked to worse CSS. Further mIF studies are necessary to explore the spectrum of CAF subpopulations in ccRCC, their interactions with other stromal cells, and their implications in the evolution of the disease.

## Supplementary Information

Below is the link to the electronic supplementary material.

Supplementary Table S1. Antibodies and conditions used for multiplexed immunophenotyping (DOCX 16 KB)

Supplementary Table S2. Association between combinations of biomarkers and their impact on cancer-specific survival (CSS) (DOCX 17 KB)

Supplementary Table S3. Univariate Cox regression analysis for cancer-specific survival (CSS) prediction in ccRCC patients (DOCX 17 KB)

Supplementary Table S4. Predictive model (Cox regression) for cancer-specific survival (CSS) prediction by combined biomarkers and pathological variables in ccRCC patients (DOCX 24 KB)

Supplementary Figure S1. Immunostaining controls for FAP specificity. Clear positivity is observed with the anti-FAP antibody (S1A), while a serial section without primary antibody shows no background staining (S1B). In this low-grade ccRCC, tumor cells are closely intermingled with stromal cells. Additional controls confirm the absence of background signal: in comparison with specific IF labeling of FAP (S1C), no signal is detected when either primary (S1D) or secondary (S1E) antibodies are omitted from the procedure (JPG 12123 KB)

Supplementary Figure S2. Control to verify the absence of fluorochrome overlap in a FAP-positive case. (S2A) mIF composite image showing all markers (CD4+, CD8+, CD4+FOXP3+, FAP+, CD68+, and CK+) merged. (S2B) The same field displaying individual channels for each marker (JPG 27589 KB)

Supplementary Figure S3. Control to verify the absence of fluorochrome overlap in a FAP-negative case. (S3A) mIF composite image with all markers (CD4+, CD8+, CD4+FOXP3+, FAP+, CD68+, and CK+) merged. (S3B) The same field displaying individual channels for each marker (JPG 29686 KB)

Supplementary Figure S4. mIF analysis of B (CD20+), T-helper (CD4+), T-cytotoxic (CD8+) and T-regulatory (CD4+FOXP3+) lymphocytes, macrophages (CD68+), and tumor cells (pan-CK+) at the center (A and C) and the periphery (B and D) of ccRCCs. In Figures B and D, the relative abundance of different stromal and tumor cells obtained through multiplex immunofluorescence is displayed (CD4+ > CD8+ > CD68+ > CD20+ > CD4+FOXP3+). A and C show the hematoxylin and eosin (H&E) staining of the same cases (JPG 39932 KB)

Supplementary Figure S5. Quantitative expression of CD20+, CD4+, CD8+, CD4+FOXP3+, and CD68+ cells at the tumor center (A) and periphery (B) of ccRCC tissues. B- and T-helper and T-cyt and T-reg lymphocytes were significantly more abundant at the tumor periphery than at the center (JPG 28982 KB)

## Data Availability

The datasets generated during and/or analyzed during the current study are available from the corresponding author on reasonable request.
