# Supplementary material for: Spatial expression of fibroblast activation protein-α in clear cell renal cell carcinomas revealed by multiplex immunoprofiling analysis of the tumor microenvironment
Source: Cancer Immunol Immunother. 2025 Jan 3;74(2):53. doi: 10.1007/s00262-024-03896-y (PMC11699175; doi:10.1007/s00262-024-03896-y)
Supplement: Supplementary file 1 — Supplementary Table S1. Antibodies and conditions used for multiplexed immunophenotyping (DOCX 16 KB) [file 262_2024_3896_MOESM1_ESM.docx]

**Supplementary Table s1.** Antibodies and conditions used for multiplexed immunophenotyping.

| **Antibodies** | **Dilution** | **Antigen retrieval** | **Opal/Dilution** |
| --- | --- | --- | --- |
| CD4 | 1:100 | Tris-EDTA, pH 9 | 520 / 1:50 |
| CD8 | 1:300 | Tris-EDTA, pH 9 | 570 / 1:50 |
| FOXP3 | 1:250 | Citrate, pH 6 | 620 / 1:50 |
| CD68 | 1:1000 | Citrate, pH 6 | 650 / 1:50 |
| CD20 | 1:250 | Citrate, pH 6 | 540 / 1:50 |
| PanCK | 1:500 | Citrate, pH 6 | 690 / 1:50 |
| FAP | 1:100 | Tris-EDTA, pH 9 | 540 / 1:50 |
