# Supplementary material for: Spatial expression of fibroblast activation protein-α in clear cell renal cell carcinomas revealed by multiplex immunoprofiling analysis of the tumor microenvironment
Source: Cancer Immunol Immunother. 2025 Jan 3;74(2):53. doi: 10.1007/s00262-024-03896-y (PMC11699175; doi:10.1007/s00262-024-03896-y)
Supplement: Supplementary file 2 — Supplementary Table S2. Association between combinations of biomarkers and their impact on cancer-specific survival (CSS) (DOCX 17 KB) [file 262_2024_3896_MOESM2_ESM.docx]

**Supplementary Table s2. Association between combinations of biomarkers and their impact on cancer-specific survival (CSS).** Double, triple and quadruple combinations of biomarkers at the tumor center (**A**) and the tumor periphery (**B**). Significant results of the Log-rank test (p < 0.05) are highlighted in bold. This indicates that when the biomarkers described in each column of the table exceeded the percentile 50 (P50) or P75 in the sample, patients' CSS was significantly worse. (*) To avoid confusion, in this table, T-regulatory cells (CD4+FOXP3+) are described as FOXP3 only.

**4A) Tumor center**

| **Percentile (P)** | **FAP/CD8** | **FAP/ FOXP3** | **FAP/CD68** | **CD8/FOXP3** | **CD8/CD68** | **FOXP3/CD68** | **FAP/CD8/FOXP3** | **FAP/CD8/CD68** | **FAP/ FOXP3/CD68** | **CD8/FOXP3/CD68** | **FAP/CD8/FOXP3/CD68** |
| --- | --- | --- | --- | --- | --- | --- | --- | --- | --- | --- | --- |
|  | Log-rank  p = | Log-rank  p = | Log-rank  p = | Log-rank  p = | Log-rank  p= | Log-rank  p = | Log-rank  p = | Log-rank  p = | Log-rank  p = | Log-rank  p = | Log-rank  p = |
| **< vs ≥ P50** | 0.214 | 0.434 | 0.056 | 0.86 | 0.67 | **0.03** | 0.501 | 0.438 | 0.228 | 0.894 | 0.601 |
| **< vs ≥ P75** | 0.216 | 0.235 | **0.038** | 0.179 | **0.005** | **0.001** | 0.389 | **0.017** | **0.002** | **0.001** | **0.016** |

**4B) Tumor periphery**

| **Percentile (P)** | **FAP/CD8** | **FAP/ FOXP3** | **FAP/CD68** | **CD8/FOXP3** | **CD8/CD68** | **FOXP3/CD68** | **FAP/CD8/FOXP3** | **FAP/CD8/ CD68** | **FAP/ FOXP3/CD68** | **CD8/FOXP3/CD68** | **FAP/CD8/FOXP3/CD68** |
| --- | --- | --- | --- | --- | --- | --- | --- | --- | --- | --- | --- |
|  | Log-rank  p = | Log-rank  p = | Log-rank  p = | Log-rank  p = | Log-rank  p= | Log-rank  p = | Log-rank  p = | Log-rank  p = | Log-rank  p = | Log-rank  p = | Log-rank  p = |
| **< vs ≥ P50** | 0.617 | 0.873 | 0.786 | 0.474 | 0.364 | 0.384 | 0.736 | 0.895 | 0.836 | 0.372 | 0.943 |
| **< vs ≥ P75** | 0.962 | 0.96 | 0.32 | 0.572 | 0.526 | 0.435 | 0.714 | 0.548 | 0.962 | 0.372 | 0.532 |
