# Supplementary material for: Spatial expression of fibroblast activation protein-α in clear cell renal cell carcinomas revealed by multiplex immunoprofiling analysis of the tumor microenvironment
Source: Cancer Immunol Immunother. 2025 Jan 3;74(2):53. doi: 10.1007/s00262-024-03896-y (PMC11699175; doi:10.1007/s00262-024-03896-y)
Supplement: Supplementary file 3 — Supplementary Table S3. Univariate Cox regression analysis for cancer-specific survival (CSS) prediction in ccRCC patients (DOCX 17 KB) [file 262_2024_3896_MOESM3_ESM.docx]

**Supplementary Table s3. Univariate Cox regression analysis for cancer-specific survival (CSS) prediction in ccRCC patients.** Selected independent variables were Percentile 50 (P50) and 75 (P75) cutoff values for the six biomarkers at both locations of the tumor, and pathological variables. ExpB with confidence interval (CI, inferior and superior) is also included. Significant results (p<0.05) are highlighted in bold.

| **Pathological Variables** | **p =** | **ExpB** | **Inf** | **Sup** |  |  |  |  |
| --- | --- | --- | --- | --- | --- | --- | --- | --- |
| **Histological Grade** (G1-G2 vs G3-G4) | **0.006** | 4.19 | 1.52 | 11.56 |  |  |  |  |
| **Necrosis** (No/Yes) | 0.11 | 2.03 | 0.84 | 4.91 |  |  |  |  |
| **Diameter** (≤ >7 cm) | **0.001** | 4.39 | 1.82 | 10.57 |  |  |  |  |
| **Local Invasion** (pT1-2 vs pT3-4) | **0.002** | 4.065 | 1.68 | 9.83 |  |  |  |  |
| **Lymph node invasion** (No/Yes) | **0.001** | 11.34 | 3.97 | 32.39 |  |  |  |  |
| **Distant Metastasis** (No/Yes) | **0.001** | 12.29 | 4.91 | 30.79 |  |  |  |  |
| **Biomarkers** |  | **Tumor center** | | |  | **Tumor periphery** | | |
|  | **p =** | **ExpB** | **Inf** | **Sup** | **p =** | **ExpB** | **Inf** | **Sup** |
| **FAP** (P50) | 0.9 | 1.06 | 0.44 | 2.54 | 0.43 | 0.69 | 0.28 | 1.73 |
| **CD4** (P50) | 0.89 | 1.07 | 0.44 | 2.56 | 0.79 | 1.13 | 0.46 | 2.79 |
| **CD8** (P50) | 0.34 | 0.65 | 0.26 | 1.59 | 0.42 | 1.46 | 0.59 | 3.63 |
| **CD20** (P50) | 0.7 | 0.84 | 0.35 | 2.03 | 0.77 | 1.14 | 0.46 | 2.81 |
| **CD4+FOXP3+** (P50) | 0.21 | 1.82 | 0.72 | 4.63 | 0.47 | 1.4 | 0.56 | 3.49 |
| **CD68** (P50) | **0.006** | 4.61 | 1.54 | 13.79 | 0.4 | 1.48 | 0.6 | 3.69 |
| **FAP** (P75) | 0.89 | 1.08 | 0.39 | 2.96 | 0.37 | 1.56 | 0.59 | 4.1 |
| **CD4** (P75) | 0.84 | 1.11 | 0.4 | 3.06 | 0.93 | 1.05 | 0.38 | 2.91 |
| **CD8** (P75) | 0.45 | 1.45 | 0.56 | 3.77 | 0.8 | 1.14 | 0.41 | 3.16 |
| **CD20** (P75) | 0.84 | 1.11 | 0.4 | 3.04 | 0.75 | 1.18 | 0.42 | 3.27 |
| **CD4+FOXP3+** (P75) | **0.037** | 2.65 | 1.06 | 6.59 | 0.97 | 1.02 | 0.37 | 2.84 |
| **CD68** (P75) | **0.001** | 5.1 | 2.11 | 12.34 | **0.033** | 2.66 | 1.08 | 6.56 |
