# Supplementary material for: Spatial expression of fibroblast activation protein-α in clear cell renal cell carcinomas revealed by multiplex immunoprofiling analysis of the tumor microenvironment
Source: Cancer Immunol Immunother. 2025 Jan 3;74(2):53. doi: 10.1007/s00262-024-03896-y (PMC11699175; doi:10.1007/s00262-024-03896-y)
Supplement: Supplementary file 4 — Supplementary Table S4. Predictive model (Cox regression) for cancer-specific survival (CSS) prediction by combined biomarkers and pathological variables in ccRCC patients (DOCX 24 KB) [file 262_2024_3896_MOESM4_ESM.docx]

**Supplementary Table s4. Predictive model (Cox regression) for cancer-specific survival (CSS) prediction by combined biomarkers and pathological variables in ccRCC patients.** Selected independent variables were double and triple combinations of biomarkers at the tumor center, and pathological variables such as Fuhrman grouped grade (low *vs* high), grouped local invasion (pT, confined *vs* not-confined), lymph node invasion (pN, no *vs* yes), and distant (pM, no *vs* yes) metastases. ExpB with confidence interval (CI, inferior and superior) is also included. Variables resulting from the backward Wald stepwise method are highlighted in bold. (*) To avoid confusion, in this table T-regulatory cells (CD4+FOXP3+) are described as FOXP3 only.

| **A** |  | **Tumor center** | | | |
| --- | --- | --- | --- | --- | --- |
| **CSS** | **Variables** | **p =** | **ExpB** | **Inf** | **Sup** |
| Multiple Cox Regression | FAP/CD68 (P75) | 0.135 | 2.57 | 0.75 | 8.84 |
|  | Grade | 0.384 | 1.70 | 0.52 | 5.58 |
|  | pT | 0.082 | 2.37 | 0.90 | 6.28 |
|  | pN | 0.161 | 2.53 | 0.69 | 9.22 |
|  | pM | 0.002 | 5.93 | 1.91 | 18.4 |
| Final Step of Wald Method | **pT** | **0.035** | 2.69 | 1.07 | 6.74 |
|  | **N** | 0.067 | 3.13 | 0.92 | 10.6 |
|  | **M** | **0.001** | 7.19 | 2.55 | 20.3 |
| **B** |  | **Tumor center** | | | |
| **CSS** | **Variables** | **p =** | **ExpB** | **Inf** | **Sup** |
| Multiple Cox Regression | CD8/CD68 (P75) | 0.983 | 1.02 | 0.24 | 4.34 |
|  | Grade | 0.424 | 1.63 | 0.49 | 5.44 |
|  | pT | 0.094 | 2.39 | 0.86 | 6.65 |
|  | pN | 0.113 | 2.90 | 0.78 | 10.8 |
|  | pM | 0.424 | 5.85 | 1.44 | 23.7 |
| Final Step of Wald Method | **pT** | **0.036** | 2.66 | 1.06 | 6.67 |
|  | **N** | 0.067 | 3.13 | 0.92 | 10.6 |
|  | **M** | **0.001** | 7.14 | 2.54 | 20.1 |
| **C** |  | **Tumor center** | | | |
| **CSS** | **Variables** | **p =** | **ExpB** | **Inf** | **Sup** |
| Multiple Cox Regression | FOXP3/CD68 (P75) | 0.026 | 4.60 | 1.21 | 17.5 |
|  | Grade | 0.331 | 1.82 | 0.54 | 6.10 |
|  | pT | 0.022 | 3.32 | 1.19 | 9.23 |
|  | pN | 0.402 | 1.75 | 0.47 | 4.48 |
|  | pM | 0.073 | 3.31 | 0.90 | 12.2 |
| Final Step of Wald Method | **FOXP3/CD68 (P75)** | **0.008** | 5.10 | 1.52 | 17.1 |
|  | **pT** | **0.007** | 4.00 | 1.47 | 10.9 |
|  | **M** | **0.005** | 4.96 | 1.61 | 15.2 |
| **D** |  | **Tumor center** | | | |
| **CSS** | **Variables** | **p =** | **ExpB** | **Inf** | **Sup** |
| Multiple Cox Regression | FOXP3/CD68 (P50) | 0.131 | 0.41 | 0.13 | 1.31 |
|  | Grade | 0.239 | 2.17 | 0.60 | 7.93 |
|  | pT | 0.113 | 2.22 | 0.83 | 5.93 |
|  | pN | 0.126 | 2.87 | 0.74 | 11.08 |
|  | pM | 0.002 | 9.45 | 2.35 | 38.0 |
| Final Step of Wald Method | **pT** | 0.060 | 2.51 | 0.96 | 6.53 |
|  | **N** | 0.075 | 3.07 | 0.89 | 10.6 |
|  | **M** | **0.001** | 7.20 | 2.50 | 20.8 |
| **E** |  | **Tumor center** | | | |
| **CSS** | **Variables** | **p =** | **ExpB** | **Inf** | **Sup** |
| Multiple Cox Regression | FAP/CD8/CD68 (P75) | 0.254 | 2.36 | 0.54 | 10.28 |
|  | Grade | 0.354 | 1.76 | 0.53 | 5.85 |
|  | pT | 0.098 | 2.27 | .86 | 6.0 |
|  | pN | 0.162 | 2.52 | 0.69 | 9.19 |
|  | pM | 0.003 | 5.66 | 1.83 | 17.5 |
| Final Step of Wald Method | **pT** | **0.035** | 2.69 | 1.07 | 6.74 |
|  | **N** | 0.067 | 3.13 | 0.92 | 10.63 |
|  | **M** | **0.001** | 7.19 | 2.55 | 20.27 |
| **F** |  | **Tumor center** | | | |
| **CSS** | **Variables** | **p =** | **ExpB** | **Inf** | **Sup** |
| Multiple Cox Regression | FAP/FOXP3/CD68 (P75) | 0.049 | 3.87 | 1.01 | 14.9 |
|  | Grade | 0.268 | 1.99 | 0.59 | 6.75 |
|  | pT | 0.124 | 2.18 | 0.81 | 5.91 |
|  | pN | 0.225 | 2.28 | 0.60 | 8.69 |
|  | pM | 0.004 | 5.44 | 1.73 | 17.2 |
| Final Step of Wald Method | **FAP/FOXP3/CD68 (P75)** | **0.026** | 4.08 | 1.18 | 14.1 |
|  | **pT** | **0.048** | 2.68 | 1.01 | 7.09 |
|  | **M** | **0.001** | 9.01 | 3.27 | 24.8 |
| **G** |  | **Tumor center** | | | |
| **CSS** | **Variables** | **p =** | **ExpB** | **Inf** | **Sup** |
| Multiple Cox Regression | CD8/FOXP3/CD68 (P75) | 0.051 | 3.52 | 0.99 | 12.4 |
|  | Grade | 0.312 | 1.88 | 0.55 | 6.39 |
|  | pT | 0.084 | 2.41 | 0.89 | 6.52 |
|  | pN | 0.104 | 2.82 | 0.81 | 10.1 |
|  | pM | 0.025 | 4.10 | 1.19 | 14.1 |
| Final Step of Wald Method | **CD8/FOXP3/CD68 (P75)** | 0.064 | 3.17 | 0.94 | 10.75 |
|  | **pT** | **0.040** | 2.75 | 1.05 | 7.23 |
|  | **N** | 0.083 | 3.11 | 0.86 | 11.17 |
|  | **M** | **0.003** | 5.5 | 1.8 | 16.8 |
